# Supplementary material for: Prevalence and predictors of work-related depression, anxiety, and stress among waiters: A cross-sectional study in upscale restaurants
Source: PLoS One. 2021 Apr 15;16(4):e0249597. doi: 10.1371/journal.pone.0249597 (PMC8049486; doi:10.1371/journal.pone.0249597)
Supplement: S1 Questionnaire — (PDF) [file pone.0249597.s001.pdf]

QUESTIONNAIRE ASSESSING DEPRESSION, ANXIETY, AND STRESS AND PREDICTORS AMONG WAITERS

**SECTION A: SOCIO-DEMOGRAPHIC INFORMATION**	
**Question**	**Response**
1. What is your sex?	Female<br>Male
2. How old are you?	[………] years
3. What is your marital status?	Single<br>Co-habiting<br>Married<br>Divorced/Separated<br>Widowed
4. Which religion do you practice?	Christianity<br>Islam<br>African Traditionalist<br>Other:…………….
5. What is your highest level of education?	Primary<br>JSS/JHS<br>SSS/SHS<br>Tertiary
6. Which ethnic group do you belong to?	Akan<br>Ewe<br>Ga/Dangme<br>Ewe<br>Mole-Dagbani<br>Other:…………..
7. For how long have you been working as a waiter?	<1 year<br>1-5 years<br>6-10 years<br>10+ years
8. For how long have you been working as a waiter?	<1 year<br>1-5 years<br>6-10 years<br>10+ years
9. What is your role in this restaurant?	Headwaiter<br>Stationed waiter<br>Supervisor

**SECTION B: PROSPECTS AND CHALLENGES ASSOCIATED WITH WAITING WORK IN UPSCALE RESTAURANTS**	
10. Are you positive about career success in current facility?	No<br>Yes
11. Do you foresee potential of extended work involvement with current facility?	No<br>Yes
12. Do you foresee better remuneration?	No

	Yes
13. Do you anticipate getting an advantage for higher roles/position in current facility?	No<br>Yes

**SECTION C: SUBSTANCE USE AMONG WAITERS**<br>0 No<br>1 Once<br>2 Twice<br>3 More than twice				
**Substance use within the last 30 days**	**0**	**1**	**2**	**3**
14. Did you consume caffeine beverage or products?				
15. Did you consume alcoholic beverage?				
16. Did you smoke cigarette smoking?				
17. Did you smoke or consume marijuana?				
18. Did you use any non-prescription drug?				

**SECTION D: SUBSTANCE USE, DEPRESSION, ANXIETY AND STRESS SCALE**<br>0 Did not apply to me at all<br>1 Applied to me to some degree, or some of the time<br>2 Applied to me to a considerable degree or a good part of time<br>3 Applied to me very much or most of the time				
**Statement**	**0**	**1**	**2**	**3**
19. I found it hard to wind down				
20. I was aware of dryness of my mouth				
21. I couldn’t seem to experience any positive feeling at all				
22. I experienced breathing difficulty (e.g. excessively rapid breathing, breathlessness in the absence of physical exertion)				
23. I found it difficult to work up the initiative to do things				
24. I tended to over-react to situations				
25. I experienced trembling (e.g. in the hands)				
26. I felt that I was using a lot of nervous energy				
27. I was worried about situations in which I might panic and make a fool of myself				
28. I felt that I had nothing to look forward to				
29. I found myself getting agitated				
30. I found it difficult to relax				
31. I felt down-hearted and blue				
32. I was intolerant of anything that kept me from getting on with what I was doing				
33. I felt I was close to panic				
34. was unable to become enthusiastic about anything				
35. I felt I wasn’t worth much as a person				
36. I felt that I was rather touchy				

37. I was aware of the action of my heart in the absence of physical exertion (e.g. sense of heart rate increase, heart missing a beat)				
38. I felt scared without any good reason				
39. I felt that life was meaningless				
